# New Insights into Microbial Diversity of the Traditional Packed Table Olives *Aloreña de Málaga* through Metataxonomic Analysis

**DOI:** 10.3390/microorganisms9030561

**Published:** 2021-03-09

**Authors:** Elio López-García, Antonio Benítez-Cabello, Javier Ramiro-García, Verónica Romero-Gil, Francisco Rodríguez-Gómez, Francisco Noé Arroyo-López

**Affiliations:** 1 Food Biotechnology Department, Instituto de la Grasa (CSIC), Campus Universitario Pablo de Olavide, Building 46. Ctra. Sevilla-Utrera, km 1, 41013 Seville, Spain; lopezgar91@gmail.com (E.L.-G.); frgomez@ig.csic.es (F.R.-G.); fnoe@ig.csic.es (F.N.A.-L.); 2 Luxembourg Centre for Systems Biomedicine, University of Luxembourg, Avenue de l’Université 2, L-4365 Esch-sur-Alzette, Luxembourg; ramiro.garcia.javier@gmail.com; 3 Technological Applications for Improvements of the Quality and Safety in Foods, Avda. Diego Martínez Barrio 10, 2ª Planta, 41013 Seville, Spain; v.romero@oleica.es

**Keywords:** shelf-life, table olives, microbial evolution, metagenomic analysis, food safety

## Abstract

*Aloreña de Málaga* is a table olive especially characterised by its natural freshness and short shelf-life. In this work, we applied a metataxonomic approach to unravel the microbial diversity of bacterial and fungi populations through the shelf-life of traditionally packed *Aloreña de Málaga*. A significant increase in lactic acid bacteria and mesophilic aerobic populations was observed during shelf-life, reaching the maximum population levels (4–5 log_10_ CFU) at the end of the study (260 days). On the contrary, a rapid reduction in yeast and mould populations was reported. The use of a metataxonomic analysis based on the amplification of 16S (bacteria) and internal transcribed spacer (ITS) region (fungi) regions revealed a low diversity for both microbial groups. *Lactiplantibacillus* (65.05 ± 8.65% in brine vs. 58.70 ± 15.70% in fruit), *Pediococcus* (28.17 ± 7.36% in brine vs. 27.20 ± 15.95% in fruit), and *Celerinatantimonas* (4.64 ± 1.08% in brine vs. 11.82 ± 18.17% in fruit) were the main genera found among bacteria, and an increase in *Lactiplantibacillus* and a reduction in *Celerinatantimonas* populations during the shelf-life were observed. On the other hand, *Citeromyces* was the dominant fungi genus (54.11 ± 2.00% in brine vs. 50.91 ± 16.14% in fruit), followed by *Candida* (8.80 ± 2.57% in brine vs. 12.32 ± 8.61% in fruit) and *Penicillium* (6.48 ± 1.87% vs. 8.48 ± 4.43% in fruit). No food-borne pathogen genera were detected in any of the samples analysed, indicating the high level of food safety found in this ready-to-eat fermented vegetable. Data obtained in this work will help in the design of new strategies for the control of microbial populations during the shelf-life of *Aloreña de Málaga*.

## 1. Introduction

Table olives are the fermented vegetables with the greatest socioeconomic importance in the Mediterranean basin. Their worldwide production nowadays exceeds 2.5 million tons/year [1]. Green Spanish-style (lye-treated olives), Californian-style (black olives darkened by oxidation), and directly brined olives (Greek-style) are the most well-known table olive elaborations [2]. The constant demand of consumers for new flavours and healthier products opens novel market niches to expand alternative olive elaborations. This is the case for *Aloreña de Málaga*, a speciality of cracked, directly brined, natural, and seasoned green olives produced in Guadalhorce Valley (Málaga, Spain). This speciality received the first Protected Designation of Origin (PDO) for table olives in Spain [3].

*Aloreña de Málaga* fruits present a characteristic green colour that is highly desirable in the final product. Presentation, characteristics, and conditions of this type of elaboration are defined in the PDO regulation [4], which recognises three kinds of presentation: (i) fresh green (packed fruits after a maximum of 3 days of fermentation), (ii) traditional (fruits fermented in brine for at least 20 days), and (iii) cured olives (fermented for at least 90 days).

In this type of table olive elaboration, it is common to avoid pasteurisation in packing to preserve the colour, texture, flavour, and natural characteristics of the product, being replaced by preservation with their physicochemical characteristics (pH, salt, and free acidity), and the use of preservatives (potassium sorbate and sodium benzoate). For this reason, they are considered as a ready-to-eat product. However, the presence of residual sugars and the use of aromatic herbs (oregano, thyme, and fennel) and seasoning material (garlic and red pepper) in the packed product, which may involve a significant supply of microorganisms [5], make it a “living product”. This means that microbiological control is essential in order to ensure a stable and safe product.

Several works have been dedicated to analysing the microorganisms present in the packed *Aloreña de Málaga* [6,7,8], which are mainly focused on the study of the product spoilage. Arroyo-López et al. [6] reported high amounts of microorganisms in the packed brine of fresh green *Aloreña de Málaga* table olives, which were higher after incorporation of seasoning material and led to a shortening of the shelf-life. Romero-Gil et al. [7] reported that spoilage of packed traditional *Aloreña de Málaga* was characterised by the formation of whitish and soft regions on the olive surface, related to a specific genotype of *Lactiplantibacillus pentosus* (ex. *Lactobacillus pentosus*). However, none of them employ culture-independent techniques, which can provide us with a more complete picture of the existing microbiota, including potentially pathogenic microorganisms, which alter the product and pose a risk to consumers.

Metagenomics techniques are excellent approaches to investigate the microbial populations present in the food matrix. Specifically, in table olives, diverse metataxonomic studies have been carried out to determine the bacteria and fungi evolution during olive fermentation [9,10,11,12,13,14]. However, few employ metataxonomic approaches to analyse both microbial groups in the final packed product. Recently, Benítez-Cabello et al. [15] used this methodology to determine the bacterial diversity present in brines of packed table olives from different regions of the world, but they did not focus their study on the fungi population.

This work aimed to determine the evolution of bacteria and fungi populations in brine and fruit samples obtained from commercial packed *Aloreña de Málaga*. A metataxonomic approach was used to evaluate the stability and food safety of this ready-to-eat fermented product.

## 2. Materials and Methods

### 2.1. Packed Aloreña de Málaga

Samples consisted of 8 different industrially packed varieties of *Aloreña de Málaga*, obtained from 2 different industries in the Guadalhorce Valley (Málaga, Spain). Olives were elaborated following the traditional style (fruits stored in brine for 20 days). In total, 16 samples were obtained from brine (B, n = 8) and fruit (F, n = 8) at different times (T0 = 0 day, T1 = 42 days, T2 = 130 days, and T3 = 260 days) during the shelf-life and analysed through microbiological, physicochemical, and amplicon massive sequencing methods. Packed *Aloreña de Málaga* were not subjected to heat treatments, so preservation was exclusively based on their physicochemical characteristics. Initial olive brine packing concentration consisted of 55 g/L NaCl, 0.8 g/L lactic acid, 1 g/L ascorbic acid, 3 g/L citric acid, 2 g/L potassium sorbate, and 1 g/L sodium benzoate. Pet packages (1.6 L total volume) were filled with 900 g of olives, 16 g of seasoning material (a mixture of diced garlic, pepper strips, and small pieces of fennel and thyme), and 0.7 L of the cover brine mentioned above. Packages were kept at room temperature (25 ± 2 °C) during analysis.

### 2.2. Physicochemical and Microbiological Analyses

The pH, free, and combined acidity values of the brines were measured using a Titroprocessor model 670 (Metrohm, Switzerland). Results of free acidity were expressed as a percentage (%, g of lactic acid per 100 mL), while combined acidity was determined as Eq/L. For the determination of NaCl concentration, Cl^−^ was titrated with silver nitrate (AgNO_3_) by mixing 0.5 mL of brine with 100 mL of distilled water. As an indicator of the reaction, a solution of potassium chromate (K_2_CrO_4_) was used. The result was expressed as a percentage (%, *w/v*) of NaCl.

First, 25 g of fruit was washed twice with sterile saline solution (0.9% *v/v*) and pitted at sterile conditions to determine the microbial biofilm formation in the olive epidermis. Then, fruit flesh was homogenised in a Stomacher^®^ (Seward Laboratory Systems, Inc., Bohemia, NY, USA) for 2 min at maximum speed, with 100 mL of sterile saline solution. Afterwards, direct or decimal dilutions of detached biofilms were plated on selective culture media. Lactic acid bacteria (LAB) were plated on the Man, Rogosa, and Sharpe (MRS) agar (Oxoid, Basingstoke, Hampshire, UK) supplemented with 0.02% sodium azide (Sigma, St. Louis, MO, USA). At the same time, *Enterobacteriaceae* were counted on crystal-violet neutral-red bile glucose (VRBD) agar (Merck, Darmstadt, Germany). Mesophilic aerobic bacteria (MAB) were plated in plate count agar (PCA) (Oxoid). Lastly, yeasts were plated in YM (yeast-mal-peptone-glucose) agar (Difco TM, Becton and Dickinson Company, Sparks, MD, USA) supplemented with gentamicin sulphate and oxytetracycline as selective agents. Plates were incubated at 37 °C in aerobic conditions for 24 and 48 h for *Enterobacteriaceae*, MAB, and LAB, respectively, and 30 °C for 48 h for yeasts. Results are expressed as log_10_ CFU/g. In the case of brine analysis, direct or decimal solutions were also spread on the selected media described above. Counts are expressed as log_10_ CFU/mL.

### 2.3. DNA Extraction from Table Olive Samples and Library Preparation

For the study of brines, 50 mL of each sample was directly taken from the packing and spun at 9000× *g* for 15 min. In the case of the fruit, 25 g of pitted olives from every sample were homogenised in 100 mL of sterile saline solution (0.9% NaCl) in a Stomacher^®^ homogeniser for 5 min and spun at 9000× *g* for 15 min. The supernatant was withdrawn, and the pellets were washed twice with sterile saline solution before storing at −80 °C until DNA extraction. The total genomic DNA from fruit and brine samples was extracted and purified using the PowerFood Microbial DNA Isolation Kit (MoBio, Carlsbad, CA, USA) according to the manufacturer’s instructions and sent for sequencing to FISABIO (Valencia, Spain). Before sequencing, purified DNA content was measured using a Qubit fluorometer (Thermo Fisher Scientific, Waltham, MA, USA), always obtaining values above 0.2 ng/µL.

For bacteria, the V3 and V4 regions (459 bp) of the 16S ribosomal RNA gene were amplified with the designed primers surrounding conserved regions [16], following the procedure described by the Illumina amplicon libraries protocol. DNA amplicon libraries were generated using a limited PCR cycle: initial denaturation at 95 °C for 3 min, followed by 25 cycles of annealing (95 °C 30 s, 55 °C 30 s, 72 °C 30 s), and a final extension at 72 °C 5 min, using a KAPA HiFi HotStart ReadyMix (KK2602).

For the fungi population, DNA samples were submitted to PCR amplification of the ITS1 region located inside the fungal nuclear ribosomal DNA (rDNA) with the designed primers surrounding conserved regions, ITS1-F_KYO2 (18S SSU 1733–1753) and ITS2_KYO2 (5.8 2046–2029) [17,18]. DNA amplicon libraries were generated using the following limited PCR cycle: initial denaturation at 95 °C for 3 min, followed by 28 cycles of annealing (95 °C 30 s, 55 °C 30 s, 72 °C 30 s), and a final extension at 72 °C 5 min, using a KAPA HiFi HotStart ReadyMix (KK2602).

Then, in both cases, the Ilumina sequencing adaptors and dual-index barcodes (Nextera XT index kit v2, FC-131-2001) were added to the amplicons. Libraries were normalised and pooled before sequencing. The pool containing indexed amplicons was loaded on the MiSeq reagent cartridge v3 (MS-102-3003) spiked with 25% PhiX control to improve base calling during sequencing, as recommended by Illumina for amplicon sequencing. Sequencing was conducted using a paired-end, 2 × 300-pb cycle run on an Illumina MiSeq sequencing system. Sequencing data have been deposited in the European Nucleotide Archive under the accession number PRJEB43241.

### 2.4. Bioinformatic Analysis

Data obtained from the 32 sequenced samples (n = 16 for bacteria and n = 16 for fungi) were analysed using NG-Tax [19] under default parameters. For each sample, only the most abundant sequences (>0.1%) were retained as Amplicon Sequence Variants (ASV); the remaining reads were clustered against those ASVs allowing one mismatch to correct for error sequencing. Taxonomy was assigned using the SILVA 138 SSURef database (containing 2,225,272 sequences) for the 16S rRNA amplicon samples (bacteria) [20] and the full UNITE+INSDC (containing 1,796,591 sequences) for the ITS amplicon samples (fungi) [21]. Alpha diversity and beta diversity indexes were calculated using R package phyloseq 1.32.0 [22] and picante 1.8.2 [23]. Plots were generated using ggplot 2 3.3.2 [24] and Metacoder 0.3.4 software packages. Finally, the tables collapsed at the genus level were exported in tab-delimited text format and statistically analysed using STAMP 2.1.3 [25]. To elucidate differences in taxa abundances between the different grouping variables, an ANOVA/Tukey–Kramer (*post hoc*) test with false discovery rate (FDR, [26]) was applied, considering significant only those differences with a *p*-value lower than 0.05 and q-value below 0.3.

## 3. Results

### 3.1. Physicochemical Analysis

**Figure 1:**
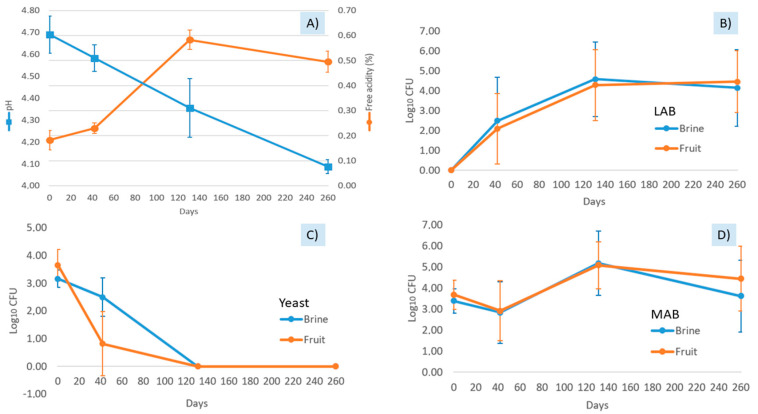
Evolution of physicochemical parameters (**A**) and microbial population (**B**–**D**) throughout the shelf-life of packed *Aloreña de Málaga*. LAB stands for lactic acid bacteria. MAB stands for mesophilic aerobic bacteria. In figure A, the blue line indicates the pH value, while free acidity is indicated as an orange line. For microbial counts, blue lines refer to brines samples, while fruit samples are indicated as orange lines. Means and error bars were obtained from two independent packed table olive samples analysed at each sampling time. No significant differences were obtained for the counts between brine and fruit matrix at the different sampling times.

Data obtained in the present study showed that physicochemical parameters were not kept constant during the shelf-life. Thus, the average pH value in brine decreased as sampling time progressed, from an initial value of 4.7 to a final value of 4.1. Consequently, free acidity increased, from an initial value of 0.20% to a final value of 0.50% (Figure 1A). Combined acidity values ranged from 0.02 to 0.06 Eq/L. Salt concentration was the only parameter that showed relative stability throughout the shelf-life, with an average value of 5.02 ± 0.85%. However, despite these physicochemical changes, we did not detect visible signs of alteration (such as swollen containers) during the 260 days of study.

### 3.2. Microbiological Analysis

The data show that traditional packed Aloreña de Málaga olives are live products due to the evolution of microbial populations. No statistical differences were observed between the counts obtained from brine and fruit ecosystems. No *Enterobacteriaceae* were detected in any of the samples analysed. On the contrary, the LAB population was not detected at the beginning of the sampling, either in brine or fruit, but the population increased up to 4 log_10_ CFU at the end of the shelf-life (Figure 1B). In the case of yeast, the initial population levels (3.5 log_10_ CFU) rapidly decreased. This was not further detected from the 120th day onwards (Figure 1C). Finally, we observed large quantities of MAB at the beginning of the packing, with 5.0 log_10_ CFU at the 130th day of shelf-life (Figure 1D). Our detection limit for viable microbial cells was 80 CFU/g or mL.

### 3.3. Metataxonomic Analysis

In the 16S rRNA amplicon study (bacteria), a total of 653,717 raw sequences were generated for the 15 samples analysed (one sample of F-T2 failed quality control), with a mean of 43,581 ± 13,323 reads per sample. After filtering ASVs, 91.1% (39,273 ± 12,023 on average) of the reads were retained. Filtered 16S sequences were assigned to 134 different bacteria ASVs. They represented 88.2 ± 3.2% of the total sample composition, while the rest were chloroplast (11.5 ± 3.2%), mitochondrial (0.1 ± 0.08%) and unclassified (0.1 ± 0.08%). On the other hand, the 14 ITS (fungi) amplicon samples analysed (one sample of F-T1 and other of F-T2 failed quality control) generated 5,164,720 raw reads, with a mean of 368,909 ± 153,327 reads per sample. After filtering ASVs, 91.7% of them (338,111 ± 141,038 on average) were retained. Filtered ITS sequences were assigned to 47 different fungi ASVs. They represented 71.3 ± 4.9% of the total sample composition, while *Bactrocera*, a fly parasite of olives, represented 2.0 ± 1.4%. Unclassified ASVs, with no correspondence in the UNITE database, accounted for 26.8 ± 4.8% of the total composition.

**Figure 2:**
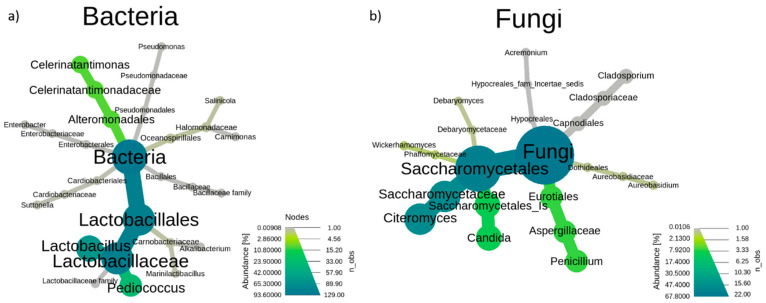
Heat tree from order to genus level obtained by 16 S (**a**) and internal transcribed spacer (ITS) region (**b**) metataxonomic analysis using the total of samples (n = 15 for bacteria and n = 14 for fungi). Samples that failed quality control (n = 3) were removed from the study. The colour and size of the branch are indicative of the abundance of sequences (%). Data were generated by grouping all sampling times.

Figure 2 shows the constructed heat tree generated from the analysis of all assigned bacteria and fungi sequences, which is indicative of the percentage of abundance obtained for the different taxonomy levels. As can be deduced from Figure 2a, *Lactobacillales* and *Alteromonadales* were the predominant orders among bacteria, the first one divided into two families, *Carnobacteriaceae* and *Lactobacillaceae*, and the second one with only a representative family, *Celerinatantimonadaceae*. Other orders such as *Oceanospirillales*, *Cardiobacteriales*, and *Enterobaterales* were also present, but all of them in a very low proportion and with only a representative family. In the fungi population, five different orders were observed, with a clear predominance of *Eurotiales* and *Saccharomycetales* (Figure 2b), the first one with a single representative family (*Aspergillaceae*)*,* and the second one with a higher proportion and three families, *Debaryomycetaceae*, *Phaffomycetaceae,* and the third one (*Sacharomycetaceae*) containing the genus *Citeromyces. Dothideales*, *Capnodiales*, and *Hyporeales* orders appeared in an almost undetectable proportion.

The fungal and bacterial diversity remained practically constant during the shelf-life of the traditional packed *Aloreña de Malaga*. As a result, alpha diversity indexes were very similar between fruit and brine ecosystems, as well as the different sampling times. As can be deduced from Table 1, no significant differences were obtained for the bacterial diversity in the Shannon index comparing brines (2.11) and olive biofilms (2.07) or for the Simpson index (0.78 brine vs. 0.75 fruit). These values were also very similar (2.08 vs. 2.11 for the Shannon index in brine and fruit, respectively, or 0.71 brine vs. 0.72 fruit for the Simpson index) regarding the diversity of fungi. Individual values for the n = 15 bacteria and n = 14 fungi samples are shown in Table S1 (see Supplementary Materials).

**Figure 3:**
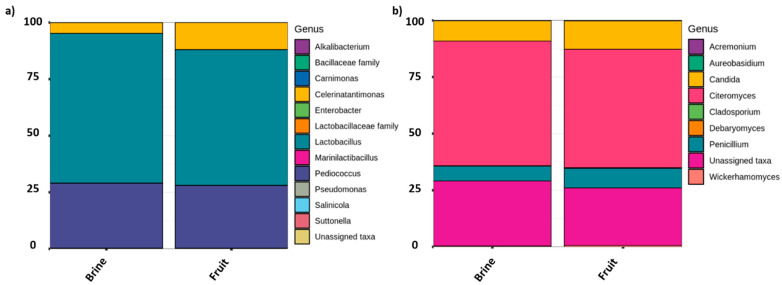
Relative abundance of sequences (%) to different taxa levels, obtained by metataxonomic analysis from 16S (**a**) and ITS (**b**) data samples of traditional packed *Aloreña de Málaga*. Values obtained for brine (n = 16) are means from n = 8 independent samples for both bacteria and fungi, while fruits (n = 13) were n = 7 for bacteria and n = 6 for fungi. Only those taxa with abundance >0.1% in at least one sample are shown. Data were generated by grouping all sampling times.

**Figure 4:**
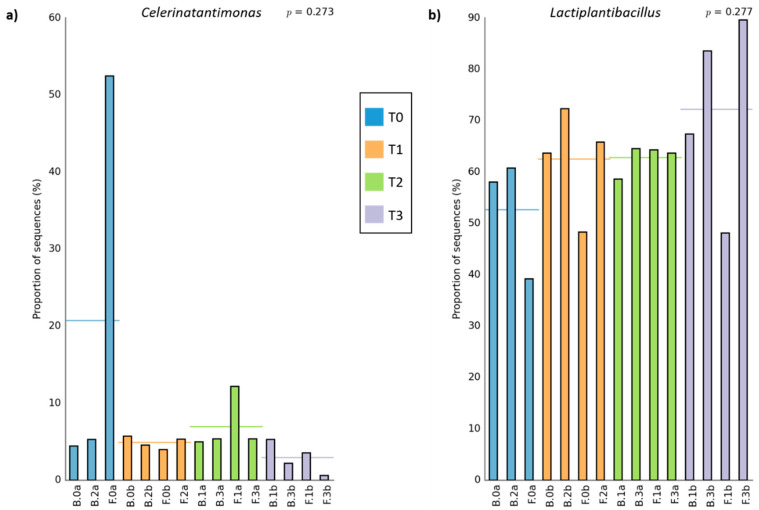
Bar plot showing the relative proportion of *Celerinatantimonas* (**a**) and *Lactiplantibacillus* (**b**) genera in the different samples of traditional packed *Aloreña de Málaga*. Horizontal lines indicate mean values (n = 3 or n = 4) for each sampling time (T0, 0 day; T1, 42 days; T2, 130 days; T3, 260 days). B stands for brines, while F stands for fruits.

As a global comparison, Figure 3 shows the relative abundance of bacteria and fungi populations at different taxa levels, grouping all samples as a function of brine or olive biofilm ecosystems. Among the bacterial populations, three main genera predominated in the packed table olives: *Lactiplantibacillus* (65.05 ± 8.65% in brine vs. 58.70 ± 15.70% in fruit), *Pediococcus* (28.17 ± 7.36% in brine vs. 27.20 ± 15.95% in fruit), and *Celerinatantimonas* (4.64 ± 1.08% in brine vs. 11.82 ± 18.17% in fruit) (Figure 3a). Statistical analysis showed that among the groups with major representation, only the presence of *Celerinatantimonas* was significantly higher in fruit than in brine samples (*p* < 0.05). As can be deduced from Figure 4a, this genus also showed a higher abundance of sequences at the beginning of the shelf-life (Figure 4a). On the other hand, Figure 4b shows that *Lactiplantibacillus* gradually increased its presence throughout the shelf-life, being significantly higher at the end of the sampling time (260 days). However, in this case, no significant differences were noticed between fruit or brine ecosystems (*p* > 0.05). Other minority bacterial groups (<0.5%) also presented significant differences between sampling times (*Alkalibacterium*, *Carnimonas*, *Enterobacter*, *Marinilactibacillus*, *Sanilicola*, and *Sutonella*). However, only *Alkalibacterium* and *Salinicola* showed significant differences between brine or fruit matrix (data not shown).

Regarding fungi diversity, three predominant genera were detected, with no significant differences between brine or fruit ecosystems (Figure 3b) or sampling time (data not shown). Thus, *Citeromyces* was the dominant fungi genus (55.02 ± 1.70% in brine vs. 52.29 ± 16.92% in fruit), followed by *Candida* (8.94 ± 2.60% in brine vs. 12.51 ± 8.54% in fruit) and *Penicillium* (6.58 ± 1.89% in brine vs. 8.63 ± 4.37% in fruit). Lastly, 27.30% of fungi sequences could not be assigned at the genera level using UNITE as the reference database.

Table 2 and Table 3 show the average relative frequency of bacteria and fungi taxa for the different combinations of olive ecosystems and sampling time. Individual values for the 29 samples are shown in Tables S2 and S3 (see Supplementary Materials). As can been deduced, no foodborne pathogen genera were detected in any sample analysed. *Salinicola* and *Marinilactibacillus* genera were also present in a high proportion of samples in the case of bacteria but with very low appearance frequencies. *Wickerhanomyces*, *Debaryomyces*, and *Aureobasidium* genera were also detected in all samples for fungi, but their presence was also reduced.

## 4. Discussion

To our knowledge, this is the first time that a combined metataxonomic analysis of the 16S and ITS sequences of fruit and brine samples has been performed for the study of packed table olives. The objective pursued after the completion of olive fermentation is to obtain a stable and safe final product. From a physicochemical point of view, packed *Aloreña de Málaga* is a safe product, as low pH values were observed once the product was stabilised, which did not permit the growth of any pathogen microorganisms. In this work, the absence of foodborne pathogens was corroborated by massive sequencing techniques. In this way, our data agree with previous challenge studies performed in commercial packed *Aloreña de Málaga* where the inhibition of different foodborne pathogens was observed [27]. Our data are also consistent with previous works that indicate that packed *Aloreña de Málaga* is a “living product”, which, due to its intrinsic properties, continues to ferment once packed [7,8,14]. The addition of seasoning material and the non-use of a heat treatment for preservation make its stabilisation challenging.

In this survey, we have reported a considerable amount of LAB and fungi in traditional packed *Aloreña de Málaga*. Regarding yeasts, the high population levels detected at the beginning of the shelf-life rapidly decreased due to the addition of potassium sorbate. Some authors had also previously reported a considerable reduction in yeast population during the shelf-life of green cracked Maçanilha table olives [28]. However, in our study, we noticed complete inhibition. Previous works have demonstrated the effectiveness of potassium sorbate to control fungi growth [29]. The yeast inhibition, together with the low salt concentration in the packed product (<6%), favoured the growth of LAB [30,31]. LAB populations’ survival and growth during the storage of diverse green table olives is well documented [32,33]. It is possible to detect a low number of LAB cells at the beginning of the shelf-life (detection limit used in this work 80 CFU) that, after acclimation, can grow.

Similar behaviour in yeasts and bacteria populations during the shelf-life of *Aloreña de Málaga* has been previously reported [7]. Although this does not pose a risk to the health of the consumer, high amounts of LAB and yeast were associated with product alterations, such as swelling containers or others, such as whitish and soft regions on the olives’ surfaces. However, no spoilage was noticed in our work, possibly since lower LAB levels were detected (4 log_10_ CFU) compared to the previous work (6 log_10_ CFU). A previous study of the instability of fresh and traditional packed *Aloreña de Málaga* olives showed that their short shelf-life (<7 days for the fresh green elaboration) was caused by the activity of microorganisms present in the packed brine, which increased after the incorporation of seasoning material [6]. Previous studies through metagenomics and traditional approaches confirmed that the seasoning material is an important source of spoilage microorganisms [5,34,35,36].

The metataxonomic analysis of the 16S region elucidated three main bacteria genera, *Lactiplantibacillus*, *Pediococcus*, and *Celerinatantimonas,* in the commercial samples. While the first two genera are the most common and responsible for the fermentation of table olives by producing lactic acid [2,37], *Celerinatantimonas* was only recently reported during table olive processing. Thus, Benítez-Cabello et al. [15] found that this genus was present in a high proportion of samples (80%) in the analysis of 72 commercially packed table olives obtained throughout the world. Other authors also reported its presence during the fermentative process of *Aloreña de Málaga* olives [12,14] and Kalamata table olives [38]. For this reason, its presence in the final product is not unusual. However, the role played by this microorganism during the fermentation or shelf-life of table olives is still unknown. Benítez-Cabello et al. [15] also pointed to *Lactobacillus* and *Pediococcus* as the most widely distributed bacteria genera found in commercially packed table olives.

The analysis of the ITS region produced approximately 26% of unassigned sequences. This may be since a significant proportion of the deposited ITS sequences are not updated or curated in the UNITE database, following the latest studies in fungal taxonomy. Despite these limits, the analysis presented sufficient resolution for most fungi genera. As in the case of bacteria, three main genera predominated throughout the shelf-life: *Candida*, *Penicillium*, and *Citeromyces*. These results are not in agreement with the data obtained by plate counts, as no yeast colonies were detected after 120 days of shelf-life. This could be due to a yeast population existing below the detection limit of the technique or yeast cells being viable but not cultivable. In this sense, it must be taken into account that metagenomic analysis is limited to the study of DNA and not RNA (metabolically active microorganisms). Despite the obvious limitations of the analysis, this omics technique is widely used in food microbiology and specifically in table olives [9,12,15,38,39], providing a fairly consistent approach to the population of microorganisms present. In our case, we analyse the relative abundance of sequences. In this way, ASVs with high abundance suggest a determining presence and function at some point in the process, to the detriment of others. Attending to the populations observed, *Candida* is one of the most common genera described during the fermentation of table olives and is responsible for producing flavours and aroma [40]. In the case of *Penicillium* and *Citeromyces*, although they are common genera during the processing of table olives, they do not usually carry significant incidence. In our study, the abundance of *Citeromyces* was higher than the rest of the yeast, with an average relative proportion of more than 50% of total sequences. Alves et al. [41] reported *Citeromyces matritensis* as one of the dominant species during cracked green table olive fermentation. Recently, Arroyo-López et al. [9] detected the presence of *Citeromyces nyonensis* in *Aloreña de Málaga* fermentations through metabarcoding analysis. For this reason, its presence in the packed product is not unusual. This genus has previously been related to other food fermentations [42]. In the case of *Penicillium*, its growth during the preservation of olives has been related to different alterations, such as the consumption of acid and the softening of olives due to the production of pectinases. On the other hand, many species of this genus can produce toxic secondary metabolites, generally known as mycotoxins [43,44,45].

The analysis also provided valuable information on the evolution and dynamics of the microbial community over time and their distribution between olive biofilms and brines in the final product. A slight but statistically significant increase in the genus *Lactiplantibacillus* was observed throughout the shelf-life, to the detriment of the rest of the bacteria and especially *Celerinatantimonas*. Simultaneously, *Celerinatantimonas* was the only predominant genus amongst all bacteria and fungi genera, showing significant differences between frequencies obtained in brine and fruit ecosystems. During the packing process, the final product is stabilised, reaching a chemical balance between the fruit and the brine, which could explain the similar results found for both olive ecosystems. Moreover, we noticed a low diversity of both bacteria and fungi genera in traditional packed *Aloreña de Málaga*. Our data agree with previous works that found a similar or even lower diversity during the shelf-life of natural table olives [15], reporting an average Simpson index value of around 0.65 for this kind of elaboration. In this sense, in the present study, we found an average value of 0.78. However, they displayed greater diversity during the shelf-life of Spanish-style table olives. The high presence of antimicrobials that characterises the elaboration and packing of directly brined olives (without NaOH treatment) could explain the lower diversity found in this table olive speciality.

## 5. Conclusions

This study highlights that packed *Aloreña de Málaga* is a living product from a physicochemical and microbiological point of view, indicating the need for better control and stabilisation of microbial populations. However, the absence of foodborne pathogen genera is a guarantee of food safety for consumers. We detected low microbial biodiversity, with scarce bacteria and fungi genera during this survey and without significant differences for the majority of them between fruit and brine ecosystems. New omics approaches based on the study of the RNA may have great relevance for a better comprehension of the activities that these microorganisms exert during the shelf-life of table olives.

## Figures and Tables

**Table 1:** Global alpha diversity indexes for bacteria (16S) and fungi (ITS) populations obtained from traditional packed *Aloreña de Málaga* table olives. Data are expressed as a mean ± the standard deviation of the values through time (T = 0 day, T1 = 42 days, T2 = 130 days, and T3 = 260 days) and olive ecosystem (brine and fruit). For bacteria of fungi populations, no significant differences were noticed between sampling times or olive ecosystems.

**Bacteria Population**					
**Time**	**Observed**	**FaithPD**	**Shannon**	**Simpson**	**Clonality**
T0 (n = 4)	42.25 ± 1.71	1.32 ± 0.09	2.09 ± 0.12	0.79 ± 0.04	0.95 ± 0.00
T1 (n = 4)	40.67 ± 2.08	1.19 ± 0.25	2.06 ± 0.06	0.78 ± 0.02	0.95 ± 0.00
T2 (n = 3)	45.00 ± 4.83	1.36 ± 0.08	2.20 ± 0.04	0.81 ± 0.01	0.95 ± 0.00
T3 (n = 4)	47.50 ± 15.78	0.98 ± 0.11	2.00 ± 0.46	0.70 ± 0.15	0.96 ± 0.01
**Matrix**					
Brine (n = 8)	42.38 ± 4.14	1.23 ± 0.19	2.11 ± 0.09	0.78 ± 0.04	0.95 ± 0.00
Fruit (n = 7)	46.00 ± 11.28	1.20 ± 0.22	2.07 ± 0.35	0.75 ± 0.12	0.95 ± 0.01
**Fungi Population**					
**Time**	**Observed**	**FaithPD**	**Shannon**	**Simpson**	**Clonality**
T0 (n = 4)	35.50 ± 1.91	2.08 ± 0.10	2.05 ± 0.09	0.70 ± 0.04	0.94 ± 0.00
T1 (n = 3)	34.67 ± 1.53	2.01 ± 0.02	2.02 ± 0.04	0.70 ± 0.01	0.94 ± 0.00
T2 (n = 3)	33.25 ± 3.40	2.07 ± 0.18	2.03 ± 0.15	0.70 ± 0.05	0.94 ± 0.00
T3 (n = 4)	32.00 ± 2.00	1.87 ± 0.22	2.29 ± 0.30	0.78 ± 0.09	0.93 ± 0.01
**Matrix**					
Brine (n = 8)	33.63 ± 2.33	1.96 ± 0.14	2.08 ± 0.07	0.71 ± 0.02	0.94 ± 0.00
Fruit (n = 6)	34.33 ± 2.94	2.09 ± 0.15	2.11 ± 0.28	0.72 ± 0.09	0.94 ± 0.01

**Table 2:** Relative abundance of sequences (%) to different taxa levels, obtained by metataxonomic analysis of 16S data samples belonging to traditional packed *Aloreña de Málaga*. Mean values were obtained from independent duplicates. Only those taxa with abundance >0.1% in at least one sample are shown.

Taxonomic Unit/Sample	* B-T0	B-T1	B-T2	B-T3	F-T0	F-T1	F-T2	F-T3
Lactiplantibacillus	58.27	66.83	60.47	74.65	43.23	47.55	64.12	62.33
Pediococcus	34.53	26.07	32.03	20.04	27.41	46.43	25.19	29.53
Celerinatantimonas	4.77	5.05	5.07	3.68	27.89	3.91	8.62	5.23
Unassigned taxa	0.23	0.11	0.19	0.15	0.09	0.12	0.19	0.19
Salinicola	0.17	0.09	0.19	0.16	0.00	0.13	0.18	0.23
Alkalibacterium	0.17	0.00	0.00	0.00	0.00	0.00	0.00	0.00
Marinilactibacillus	0.00	0.14	0.08	0.08	0.06	0.17	0.15	0.19
Bacillaceae family	0.00	0.00	0.12	0.00	0.00	0.00	0.00	0.00
Enterobacter	0.00	0.00	0.00	0.00	0.18	0.00	0.00	0.00
Pseudomonas	0.00	0.00	0.00	0.00	0.12	0.00	0.00	0.00
Suttonella	0.00	0.00	0.06	0.08	0.00	0.00	0.14	0.17
Carnimonas	0.00	0.00	0.00	0.00	0.11	0.00	0.00	0.00
Lactobacillaceae family	0.00	0.00	0.00	0.00	0.00	0.15	0.00	0.00

* B = Brine, F = fruit. (T0 = 0 day, T1 = 42 days, T2 = 130 days, and T3 = 260 days).

**Table 3:** Relative abundance of sequences (%) to genus level, obtained by metataxonomic analysis of ITS data samples belonging to traditional packed *Aloreña de Málaga*. Mean values were obtained from independent duplicates. Only those genera with abundance >0.1% in at least one sample are shown.

Genus/Sample	* B-T0	B-T1	B-T2	B-T3	F-T0	F-T1	F-T2	F-T3
Citeromyces	53.63	56.46	56.05	53.95	57.95	57.63	56.88	19.25
Unassigned taxa	27.24	27.06	26.13	34.53	24.03	24.53	24.14	32.01
Candida	10.31	9.17	9.92	6.36	10.17	10.01	10.17	29.20
Penicillium	7.89	6.57	7.07	4.79	7.00	6.46	7.76	17.01
Wickerhamomyces	0.52	0.31	0.46	0.18	0.48	0.53	0.45	1.86
Aureobasidium	0.23	0.18	0.21	0.12	0.22	0.21	0.28	0.00
Debaryomyces	0.18	0.08	0.16	0.08	0.16	0.17	0.19	0.46
Cladosporium	0.00	0.00	0.00	0.00	0.00	0.00	0.13	0.00

* B = Brine, F = fruit. (T0 = 0 day, T1 = 42 days, T2 = 130 days, and T3 = 260 days).

## Data Availability

Sequencing data have been deposited in the European Nucleotide Archive (https://www.ebi.ac.uk/ena/browser/home) under the accession number PRJEB43241.

## Supplementary Materials

The following are available online at https://www.mdpi.com/2076-2607/9/3/561/s1, Table S1: Alpha diversity indexes for bacteria (16S) and fungi (ITS) populations obtained from packed traditional *Aloreña de Málaga* table olives, Table S2: Relative abundance (%) of bacteria (16S) obtained from packed traditional *Aloreña de Málaga* table olives (n = 15). Only those genus/family with abundance >0.1% in at least one sample are shown, Table S3: Relative abundance (%) of fungi (ITS) obtained from packed traditional *Aloreña de Málaga* table olives (n = 14). Only those genus with abundance >0.1% in at least one sample are shown.

## References

[1] International Olive Oil Council Economic Affairs & Promotion Unit-International Olive Council. https://www.internationaloliveoil.org/what-we-do/economic-affairs-promotion-unit/#figures.

[2] Garrido-Fernández A., Fernández Díez M.J., Adams R.M. (1997). Table Olives: Production and Processing.

[3] Regional Government of Andalusia (2009). Order Approving the Application for Registration of the Protected Designation of Origin “Aceituna Aloreña de Málaga”.

[4] Official Journal of the European Union (DOUE) Regulation Nº1068/2012. L318/3-L318/4. https://www.boe.es/buscar/doc.php?id=DOUE-L-2012-82202.

[5] Benítez-Cabello A., Romero-Gil V., Medina E., Sánchez B., Calero-Delgado B., Bautista-Gallego J., Jiménez-Díaz R., Arroyo-López F.N. (2019). Metataxonomic analysis of the bacterial diversity in table olive dressing components. Food Control.

[6] Arroyo-López F.N., Bautista-Gallego J., Segovia-Bravo K.A., García-García P., Durán-Quintana M.C., Romero C., Garrido-Fernández A. (2009). Instability profile of fresh packed “seasoned” Manzanilla-Aloreña table olives. Food Sci. Technol..

[7] Romero-Gil V., Rodríguez-Gómez F., Garrido-Fernández A., García-García P., Arroyo-López F. (2016). *Lactobacillus pentosus* is the dominant species in spoilt packaged Aloreña de Málaga table olives. LWT.

[8] Romero-Gil V., Rodríguez-Gómez F.J., Ruiz-Bellido M.A., Benítez-Cabello A., Garrido Fernández A., Arroyo López F.N. (2019). Shelf-life of traditionally-seasoned Aloreña de Málaga table olives based on package appearance and fruit characteris-tics. Grasas Aceites.

[9] Arroyo-López F.N., Medina E., Ruiz-Bellido M.Á., Romero-Gil V., Montes-Borrego M., Landa B.B. (2016). Enhancement of the knowledge on fungal communities in directly brined aloreña de málaga green olive fermentations by metabarcoding analysis. PLoS ONE.

[10] Cocolin L., Alessandria V., Botta C., Gorra R., De Filippis F., Ercolini D., Rantsiou K. (2013). NaOH-debittering induces changes in bacterial ecology during table olives fermentation. PLoS ONE.

[11] De Angelis M., Campanella D., Cosmai L., Summo C., Rizzello C.G., Caponio F. (2015). Microbiota and metabolome of un-started and started Greek-type fermentation of Bella di Cerignola table olives. Food Microbiol..

[12] Medina E., Bellido M.A.R., Romero-Gil V., Rodríguez-Gómez F.J., Montes-Borrego M., Landa B.B., López F.N.A. (2016). Assessment of the bacterial community in directly brined Aloreña de Málaga table olive fermentations by metagenetic analysis. Int. J. Food Microbiol..

[13] Medina E., Brenes M., García-García P., Romero C., de Castro A. (2018). Microbial ecology along the processing of Spanish olives darkened by oxidation. Food Control.

[14] Rodríguez-Gómez F.J., Ruiz-Bellido M.Á., Romero-Gil V., Benítez-Cabello A., Garrido-Fernández A., López F.N.A. (2017). Microbiological and physicochemical changes in natural green heat-shocked aloreña de málaga table olives. Front. Microbiol..

[15] Benítez-Cabello A., Romero-Gil V., Medina-Pradas E., Garrido-Fernández A., Arroyo-López F.N. (2020). Exploring bacteria diversity in commercialized table olive biofilms by metataxonomic and compositional data analysis. Sci. Rep..

[16] Klindworth A., Pruesse E., Schweer T., Peplies J., Quast C., Horn M., Glöckner F.O. (2012). Evaluation of general 16S ribosomal RNA gene PCR primers for classical and next-generation sequencing-based diversity studies. Nucleic Acids Res..

[17] Toju H., Tanabe A.S., Yamamoto S., Sato H. (2012). High-coverage ITS primers for the DNA-based identification of as-comycetes and basidiomycetes in environmental samples. PLoS ONE.

[18] Gardes M., Bruns T.D. (1993). ITS primers with enhanced specificity for basidiomycetes-application to the identification of mycorrhizae and rusts. Mol. Ecol..

[19] Ramiro-Garcia J., Hermes G.D., Giatsis C., Sipkema D., Zoetendal E.G., Schaap P.J., Smidt H. (2018). NG-Tax, a highly accurate and validated pipeline for analysis of 16S rRNA amplicons from complex biomes. F1000Research.

[20] Quast C., Pruesse E., Yilmaz P., Gerken J., Schweer T., Yarza P., Glöckner F.O. (2012). The SILVA ribosomal RNA gene database project: Improved data processing and web-based tools. Nucleic Acids Res..

[21] Abarenkov K., Zirk A., Piirmann T., Pöhönen R., Ivanov F., Nilsson R., Kõljalg U. (2020). Full UNITE+INSD Dataset for Eukaryotes. https://search.datacite.org/works/10.15156/bio/786373.

[22] McMurdie P.J., Holmes S. (2013). Phyloseq: An R package for reproducible interactive analysis and graphics of microbiome census data. PLoS ONE.

[23] Kembel S.W., Cowan P.D., Helmus M.R., Cornwell W.K., Morlon H., Ackerly D.D., Blomberg S.P., Webb C.O. (2010). Picante: R tools for integrating phylogenies and ecology. Bioinformatics.

[24] Wickham H. (2016). ggplot2: Elegant Graphics for Data Analysis.

[25] Parks D.H., Tyson G.W., Hugenholtz P., Beiko R.G. (2014). STAMP: Statistical analysis of taxonomic and functional pro-files. Bioinformatics.

[26] Benjamini Y., Hochberg Y. (1995). Controlling the false discovery rate—A practical and powerful approach to multiple testing. J. R. Stat. Soc. Ser. B Methodol..

[27] Romero-Gil V., Medina E., Garrido-Fernández A., Arroyo-López F.N. (2018). Foodborne pathogen survival in comercial Aloreña de Málaga table olive packaging. Front. Microbiol..

[28] Alves M., Esteves E., Quintas C. (2015). Effect of preservatives and acidifying agents on the shelf life of packed cracked green table olives from Maçanilha cultivar. Food Packag. Shelf Life.

[29] Arroyo-López F., Querol A., Bautista-Gallego J., Garrido-Fernández A. (2008). Role of yeasts in table olive production. Int. J. Food Microbiol..

[30] López F.N.A., Romero C., Quintana M.D.C.D., López A.L., García P.G., Fernández A.G. (2005). Kinetic study of the physicochemical and microbiological changes in “Seasoned” olives during the shelf-life period. J. Agric. Food Chem..

[31] López F.N.A., Quintana M.C.D., Fernández A.G. (2006). Microbial evolution during storage of seasoned olives prepared with organic acids with potassium sorbate, sodium benzoate, and ozone used as preservatives. J. Food Prot..

[32] Casado F.J., Sánchez A.H., De Castro A., Rejano L., Beato V.M., Montaño A. (2011). Fermented vegetables containing benzoic and ascorbic acids as additives: Benzene formation during storage and impact of additives on quality parameters. J. Agric. Food Chem..

[33] Blana V.A., Polymeneas N., Tassou C.C., Panagou E.Z. (2016). Survival of potential probiotic lactic acid bacteria on fermented green table olives during packaging in polyethylene pouches at 4 and 20 °C. Food Microbiol..

[34] Baumgartner A., Grand M., Liniger M., Iversen C. (2009). Detection and frequency of *Cronobacter* spp. (*Enterobacter sa-kazakii*) in different categories of ready-to-eat foods other than infant formula. Int. J. Food Microbiol..

[35] Beuchat L.R., Komitopoulou E., Beckers H., Betts R.P., Bourdichon F., Fanning S., Joosten H.M., Ter Kuile B.H. (2013). Low–Water activity foods: Increased concern as vehicles of foodborne pathogens. J. Food Prot..

[36] Vij V., Ailes E., Wolyniak C., Angulo F.J., Klontz K.C. (2006). Recalls of spices due to bacterial contamination monitored by the U.S. food and drug administration: The predominance of *Salmonellae*. J. Food Prot..

[37] Hurtado A., Reguant C., Bordons A., Rozès N. (2012). Lactic acid bacteria from fermented table olives. Food Microbiol..

[38] Kazou M., Tzamourani A., Panagou E.Z., Tsakalidou E. (2020). Unraveling the microbiota of natural black cv. Kalamata fermented olives through 16S and ITS metataxonomic analysis. Microorganisms.

[39] Argyri K., Doulgeraki A.I., Manthou E., Grounta A., Argyri A.A., Nychas G.J.E., Tassou C.C. (2020). Microbial di-versity of fermented greek table olives of halkidiki and konservolia varieties from different regions as revealed by metagenomic analysis. Microorganisms.

[40] Arroyo-López F., Romero-Gil V., Bautista-Gallego J., Rodríguez-Gómez F., Jiménez-Díaz R., García-García P., Querol A., Garrido-Fernández A. (2012). Yeasts in table olive processing: Desirable or spoilage microorganisms?. Int. J. Food Microbiol..

[41] Alves M., Gonçalves T., Quintas C. (2012). Microbial quality and yeast population dynamics in cracked green table olives’ fermentations. Food Control.

[42] Kurtzman C., Fell J.W., Boekhout T. (2011). The Yeasts: A Taxonomic Study.

[43] Heperkan D., Meric B.E., Sismanoglu G., Dalkiliç G., Güler F.K. (2006). Mycobiota, Mycotoxigenic Fungi, and Citrinin Production in Black Olives. Advances in Food Mycology.

[44] Heperkan D., Dazkır G.S., Kansu D.Z., Karbancıoglu Güler F. (2009). Influence of temperature on citrinin accumulation by *Penicillium citrinum* and *Peniccillium verrucosum* in black table olives. Toxin Rev..

[45] Medina-Pradas E., Arroyo-López F.N. (2015). Presence of toxic microbial metabolites in table olives. Front. Microbiol..

